# Identification of gene signatures associated with ulcerative colitis and the association with immune infiltrates in colon cancer

**DOI:** 10.3389/fimmu.2023.1086898

**Published:** 2023-01-19

**Authors:** Zhaoji Pan, Hao Lin, Yanyan Fu, Fanpeng Zeng, Feng Gu, Guoping Niu, Jian Fang, Bing Gu

**Affiliations:** ^1^ Department of Clinical Laboratory, Xuzhou Central Hospital, Xuzhou Institute of Medical Sciences, Xuzhou Clinical School of Xuzhou Medical University, Xuzhou, Jiangsu, China; ^2^ Department of Gastrointestinal Surgery, Xuzhou Central Hospital, Xuzhou Clinical School of Xuzhou Medical University, Xuzhou, Jiangsu, China; ^3^ Department of Cell Biology and Neurobiology, Xuzhou Medical University, Xuzhou, Jiangsu, China; ^4^ Department of Blood Transfusion, The First Affiliated Hospital of Anhui Medical University, Hefei, Anhui, China; ^5^ Laboratory Medicine, Guangdong Provincial People’s Hospital, Guangdong Academy of Medical Sciences, Guangzhou, Guangdong, China

**Keywords:** UC, GEO dataset, biomarkers, colon cancer, immune

## Abstract

**Background:**

Inflammatory bowel diseases, including ulcerative colitis (UC) and Crohn’s disease, are some of the most common inflammatory disorders of the gastrointestinal tract. The dysfunction of the immune system in the intestines is suggested to be the underlying cause of the pathogenesis of UC. However, the mechanisms regulating these dysfunctional immune cells and inflammatory phenotypes are still unclear.

**Methods:**

The differential expression analysis on microarray datasets were performed including GSE24287, GSE87466, GSE102133, and GSE107499, including 376 samples. “Gene Ontology” and “Kyoto Encyclopedia of Genes and Genomes” pathway enrichment analyses were conducted to identify the common differentially expressed genes (DEGs) in these datasets and explore their underlying biological mechanisms. Further algorithms like “Cell-type Identification by Estimating Relative Subsets of RNA Transcripts” were used to determine the infiltration status of immune cells in patients with UC. “Cytoscape” and “Gene Set Enrichment Analysis” were used to screen for hub genes and to investigate their biological mechanisms. The Tumor Immune Estimation Resource database was used to study the correlation between hub genes and infiltrating immune cells in patients with UC. A total of three hub genes, CCL3, MMP3, and TIMP1, were identified using Cytoscape.

**Results:**

A positive correlation was observed between these hub genes and patients with active UC. These genes served as a biomarker for active UC. Moreover, a decrease in CCL3, MMP3, and TIMP1 expression was observed in the mucosa of the intestine of patients with active UC who responded to Golimumab therapy. In addition, results show a significant positive correlation between CCL3, MMP3, and TIMP1 expression and different immune cell types including dendritic cells, macrophages, CD8+ T cells, and neutrophils in patients with colon cancer. Moreover, CCL3, MMP3, and TIMP1 expression were strongly correlated with different immune cell markers.

**Conclusion:**

Study results show the involvement of hub genes like CCL3, MMP3, and TIMP1 in the pathogenesis of UC. These genes could serve as a novel pharmacological regulator of UC. These could be used as a therapeutic target for treating patients with UC and may serve as biomarkers for immune cell infiltration in colon cancer.

## 1 Introduction

Inflammatory bowel diseases (IBDs) and their two primary subtypes, Crohn’s disease (CD) and ulcerative colitis (UC), cause digestive disorders and inflammation in the gastrointestinal tract. UC is a chronic recurrent IBD characterized by abdominal cramps, pus, or mucinous stools. The lesion usually originates in the rectum, continues to the proximal colon, and is often confined to the mucosal layer (1, 2). The course of the disease in patients with UC is long, has a higher chance of relapse, and is challenging to treat. Patients with UC have a higher risk of developing colorectal cancer (3). World Health Organization has classified UC as a refractory disease that seriously threatens human health (4, 5).

However, the pathogenesis of UC is complex. Studies have shown the involvement of multiple factors such as environmental and psychological factors, epithelial barrier defects, and immune disorders, including abnormal cytokine secretion and immune response dysregulation in the pathogenesis of UC (6, 7). Several clinical indicators/biomarkers used for diagnosing and monitoring UC lack sensitivity and specificity in distinguishing UC from other functional intestinal diseases (8). Patients with UC usually undergo the active and remission phases. The goals for successful treatment of UC include early remission and long-term maintenance to prevent recurrences. Currently, the commonly used drugs for treating patients with UC include glucocorticoids, immunomodulators, aminosalicylate, biological agents, etc. However, few biomarkers can accurately predict drug sensitivity/resistance in patients with UC. Failure of the immune tolerance mechanism or damaged normal intestinal barrier leads to excessive infiltration of immune cells in lamina propria of the intestine, which is then exposed to invading luminal antigens (9). These activated immune cells secrete chemokines and cytokines including interferon-γ, interleukins, and tumor necrosis factor (TNF) to promote inflammation (10). Studies have shown that an imbalance between inflammation and mucosal immunity is an important feature of colorectal cancer, which also increases the risk of colorectal cancer progression in patients with UC (11, 12). Therefore, it is necessary to determine the molecular basis and mechanism of the pathogenesis of UC and identify specific biomarkers to diagnose and treat patients with UC. This will help address the problem related to the diagnosis and treatment of patients with UC.

Therefore, in this study, we retrieved datasets like GSE87466, GSE107499, GSE24287, and GSE102133 related to IBD from multiple databases like Gene Expression Omnibus (GEO, http://www.ncbi.nih.gov/geo) (13–15). We used algorithms and databases like “Gene Set Enrichment Analysis (GSEA),” “Gene Ontology (GO),” and “Kyoto Encyclopedia of Genes and Genomes (KEGG) pathway” enrichment analyses to determine the potential underlying mechanism. We also used the “Cell-type Identification by Estimating Relative Subsets of RNA Transcripts (CIBERSORT)” algorithm to determine the proportion of 22 immune cell types in tissues of patients with UC. We generated Venn diagrams to identify differentially expressed genes (DEGs). Finally, we identified three key hub genes using Cytoscape. Further, the Tumor Immune Estimation Resource (TIMER) and Gene Expression Profiling Interactive Analysis (GEPIA) databases were used to determine the potential correlation between the expression of three hub genes and the level of immune cell infiltration in colon cancer.

## 2 Materials and methods

### 2.1 Patient selection and tissue collection

Immunohistochemistry (IHC) was performed on 20 UC tissues and paired adjacent normal tissues collected between January 2022 and December 2022 from Xuzhou Central Hospital, Jiangsu, China. All experiments were conducted in accordance with the Declaration of Helsinki. Written informed consent was obtained from all patients.

### 2.2 IHC

IHC was performed as describe previously (16). Briefly, the tissues were exposed to primary antibodies like macrophage inflammatory protein-1α (CCL3; 1:200, DF8572, Affinity), MMP3 Monoclonal antibody (1:200, 66338-1-Ig, Proteintech), and TIMP1 polyclonal antibody (1:200, 16644-1-AP, Proteintech). The tissues were stained with hematoxylin-eosin (HE) using the HE staining Kit (KGA224, keyGEN BioTECH, Nanjing, Jiangsu).

### 2.3 Cell culture

Normal colon mucosa NCM460 cells were cultured in Dulbecco’s modified Eagle’s medium (DMEM; KGM31600; keyGEN BioTECH, Nanjing, Jiangsu.) containing 10% fetal bovine serum. 1 X 105 NCM460 cells were cultured in 6-well plates and stimulated by 1 ug LPS for 12 h and 24 h at 37°C. Proinflammatory cytokines IL-1β and IL-8 in the supernatants of different treatment groups were measured by Flow cytometry.

### 2.4 Western blotting

Western blotting was performed as described previously (16). The membranes/blot were incubated with primary antibodies like CCL3, MMP3, TIMP1, and GAPDH (CCL3; 1:1000, DF8572, Affinity; MMP3 Monoclonal antibody, 1:5000, 66338-1-Ig, Proteintech; TIMP1 polyclonal antibody, 1:1000, 16644-1-AP, Proteintech; GAPDH, 1:20000, 60004-1-Ig, Proteintech; Rainbow 180 plus Marker, 39.VPM003, VICMED; Universal antibody diluent, 39.VP6022-50, VICMED). Next, the membranes/blots were incubated with HRP-conjugated secondary antibody for 1 h. We used enhanced chemiluminescence (ECL) system (Image Quant LAS 4000 mini, Pittsburgh, USA) for detecting the protein bands as per instructions. All experiments were performed in triplicates.

### 2.5 Data acquisition and DEG analysis

The microarray datasets like GSE87466, GSE107499, GSE24287, and GSE102133 were downloaded from GEO as MINiML files. The normalized quantiles functions of the “preprocessCore” R package (version 3.4.1) were used to normalize the microarray data. A principal component analysis plot was constructed to characterize the samples before and after removing the batch effect (17). The“linear models for microarray data” and “RobustRankAggreg” R package were used to identify DEGs and statistically changed genes (adj. p-value < 0.05). Finally, we constructed a Venn diagram to identify the common DEGs between the datasets.

### 2.6 Functional enrichment analysis of DEGs and hub genes

GO enrichment analysis was used to annotate gene sets into the biological process (BP), molecular function (MF), and cellular component (CC) terms. The KEGG pathway enrichment analysis was conducted to identify the signaling pathways related to DEGs. The Metascape database (https://metascape.org/gp/index.html#/main/step1) was used to perform GO and KEGG pathway enrichment analyses (18). p < 0.05 was set as the criterion to identify the significantly enriched GO terms and KEGG pathways. Additionally, GSEA was performed to study the underlying biological mechanisms of UC, and the results were calculated with the aid of Sangerbox3.0 (http://sangerbox.com/home.html), a web-based portal (19). We divided the samples based on hub gene expression into the high (≥ 50%) and low (< 50%) expression groups to identify the pathways and molecular mechanisms associated with UC.

### 2.7 Determining the infiltration of immune cells in UC

The “CIBERSORT” algorithm is a gene expression-based deconvolution method to determine the cellular composition of a tissue using the LM22 reference set. The “CIBERSORT” algorithm results were calculated using Sangerbox3.0 (19).

### 2.8 Animal model of colitis

Tissues from the Dextran sulfate sodium (DSS)-induced colitis mouse model were donated by Dr. Zhao Mingxia, School of Basic Medicine, Anhui Medical University. All procedures involving animals were reviewed and approved by the Ethics Committee of Anhui Medical University (No. 20180353).

### 2.9 Statistical analysis

The Wilcoxon signed-rank test and the Kruskal-Wallis test were used for performing the expression analysis in patients with UC and control individuals. The correlation between the expression of CCL3, MMP3, and TIMP1 and immune cells in tumors was determined using Spearman correlation. The strength of the correlation was represented as R-value: where 0–0.19 denoted “very weak,” 0.20–0.39 denoted “weak,” 0.40–0.59 denoted “moderate,” 0.60–0.79 denoted “strong,” 0.80–1.00 denoted “very strong.” *P* < 0.05 were considered statistically significant. * *P* < 0.05, ** P < 0.01, *** *P* < 0.001.

## 3 Results

### 3.1 Identification of DEGs, functional annotation, and hub gene selection

**Figure 1:**
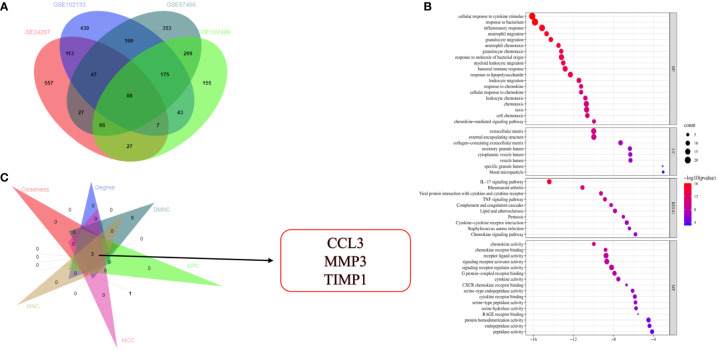
Identification and functional annotation of DEGs and hub gene selection. **(A)** Venn diagram shows overlapping genes from four gene sets. **(B)** GO and KEGG pathway enrichment analysis of DEGs. **(C)** Identification of three hub genes.

Gene expression data obtained from four microarray datasets, GS87466, GSE107499, GSE24287, and GSE102133, were used for differential expression analysis. We identified 88 DEGs (**Figure 1A**). These 88 DEGs were subjected to “GO and KEGG enrichment analyses” to identify the potential functions and underlying mechanisms associated with IBD. Most GO terms of biological process (BP) were focused on the inflammatory response, cell migration, as well as chemotaxis of neutrophils and granulocytes (**Figure 1B**). GO terms of molecular function (MF) were focused on the chemokine activity. Moreover, DEGs were enriched in pathways like the IL-17 and TNF signaling pathways and cytokine-cytokine receptor interaction (**Figure 1B**). The STRING database (http://string-db.org) was used for predicting the protein-protein interaction (PPI) network of target genes. The results were visualized using Cytoscape (version 3.7.2). Next, a Cytoscape plug-in like “Molecular Complex Detection” was used to identify and visualize important modules of the densely connected nodes in the PPI network of target genes. Cytoscape plug-in, “cytoHubba”, was used to identify the key genes in the PPI network. We identified the top 20 genes by the intersection of overlapping genes from six algorithms like “Edge Percolated Component,” “Maximal Clique Centrality,” “Closeness,” “BottleNeck,” “Degree,” and “Maximum Neighborhood Component.” The results indicated three genes, excluding CCL3, MMP3, and TIMP1 (**Figure 1C**).

### 3.2 Immune-infiltrating landscape of UC and biological processes in the hub genes

**Figure 2:**
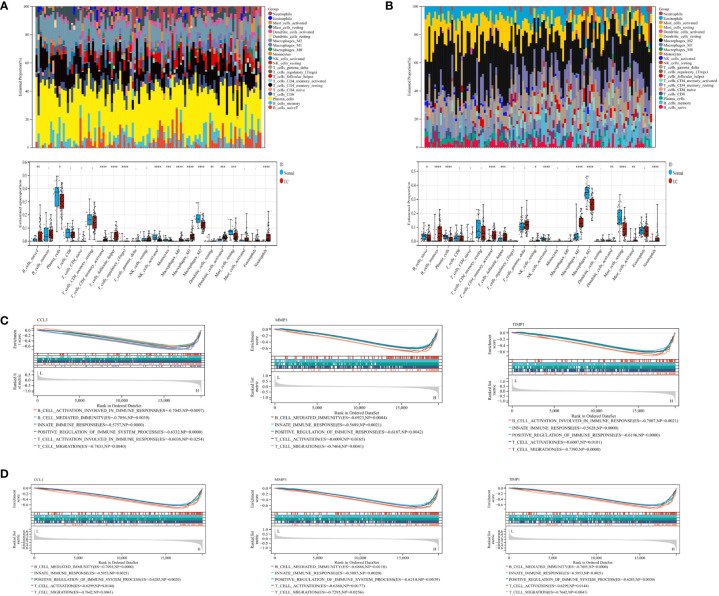
CIBERSORT was used to determine immune cell infiltration status in samples from the GSE87466 and GSE107499 datasets. GSEA was performed on CCL3, MMP3, and TIMP1 to identify biological processes. **(A)** Stacked bar plots and box plots show the relative levels of 22 immune cells in the GSE87466 dataset. **(B)** Stacked bar plots and boxplots show the relative levels of 22 immune cells in the GSE107499 dataset. **(C, D)** GSEA shows the biological processes related to CCL3, MMP3, and TIMP1 in GSE87466 and GSE107499. "*" corresponds to a significant level of 5%, that is, p<0.05. p<0.05 indicates that the difference is 95% certain and has statistical significance. "**" corresponds to a significant level of 1%, that is, p<0.01, which indicates that the difference is 99% certain, and the difference is significant, with statistical significance. "***" corresponds to a significant level of 0.1%, that is, p<0.001. p<0.001 indicates that the difference is 99.9%, which is very significant and statistically significant.

The pathogenesis of UC may be associated with immune dysregulation in the mucosa of the intestine in patients with UC. Therefore, to understand the pathogenesis of UC, we used CIBERSORT for comparing the differences in the level of 22 immune cell types infiltrating in the intestinal mucosa of the healthy controls and patients with UC from the GSE87466 (**Figure 2A**) and GSE107499 (**Figure 2B**) datasets. The results revealed an increase in the level of infiltration of immune cells like T follicular helper cells, M1 macrophages, neutrophils, and memory-activated CD4^+^ T cells, in the intestinal mucosa of patients with UC compared to healthy controls. Moreover, high infiltration levels of immune cells like plasma cells, active NK cells, and M2 macrophages were observed in the intestinal mucosa of healthy controls. GSEA was performed to study the potential biological processes associated with hub genes like CCL3, MMP3, and TIMP1. The results showed that these hub genes were associated with processes like innate immune response, positive regulation of the immune system, B cell mediated-immune responses, and T cell activation migration in the GSE87466 (**Figure 2C**) and GSE107499 (**Figure 2D**) datasets. Together, this indicates that these hub genes mediated immune response during the disease process in patients with UC.

### 3.3 Verification of related hub gene expression

**Figure 3:**
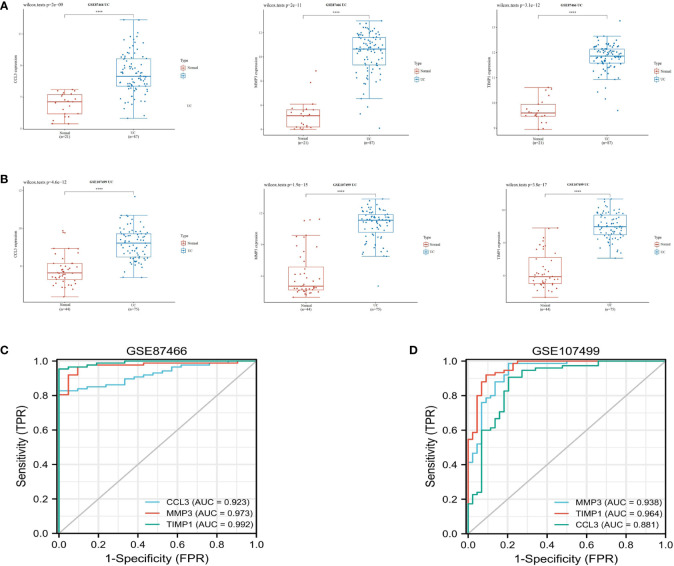
Verification of hub genes. **(A, B)** The expression of CCL3, MMP3, and TIMP1 in GSE87466 and GSE107499. **(C, D)** ROC analysis of CCL3, MMP3, and TIMP1 in the GSE87466 and GSE107499 datasets. "****" corresponds to 0.01% significant level, that is, p<0.0001, p<0.0001, which indicates that the difference is 99.99% certain, and the difference is very significant, with statistical significance.

**Figure 4:**
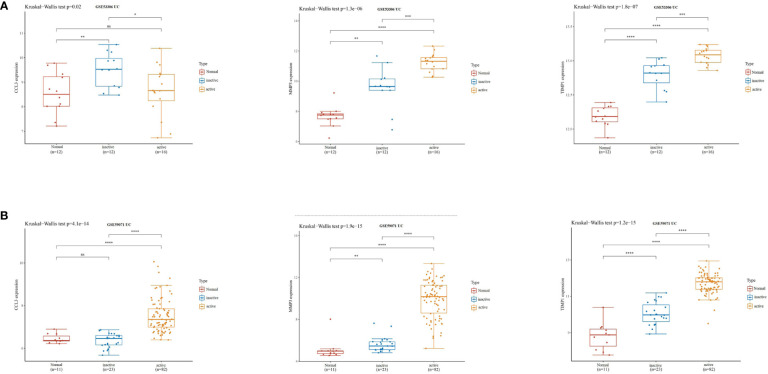
CCL3, MMP3, and TIMP1 expression levels in patients with active UC. **(A)** The expression of CCL3, MMP3, and TIMP1 in the GSE53306 dataset. **(B)** The expression of CCL3, MMP3, and TIMP1 in the GSE59071 dataset. "*" corresponds to a significant level of 5%, that is, p<0.05. p<0.05 indicates that the difference is 95% certain and has statistical significance. "**" corresponds to a significant level of 1%, that is, p<0.01, which indicates that the difference is 99% certain, and the difference is significant, with statistical significance. "***" corresponds to a significant level of 0.1%, that is, p<0.001. p<0.001 indicates that the difference is 99.9%, which is very significant and statistically significant. ns means no statistical significance.

Subsequently, we validated the differential expression of these three hub genes in the GSE87466 and GSE107499 datasets. The results indicated an increase in CCL3, MMP3, and TIMP1 expression in the colon tissue lesions of patients with UC (**Figures 3A, B**). The receiver operating characteristic (ROC) analysis was performed on these three hub genes based on the GSE87466 and GSE107499 datasets. The results revealed that the performance of these three genes in predicting the diagnosis of patients with UC was good. The area under the ROC curve value of these three hub genes was relatively high: 0.923, 0.973, and 0.992 in GSE87466 (**Figure 3C**) and 0.881, 0.938, and 0.964 in GSE107499 (**Figure 3D**), respectively. Furthermore, we determine CCL3, MMP3, and TIMP1 expression in patients with active UC from the GSE53306 (20) and GSE59071 (21) datasets. **Figures 4A, B** show the differences in the expression of CCL3, MMP3, and TIMP1 across healthy controls, inactive as well as active intestinal mucosal tissues.

### 3.4 Validation of the expression of CCL3, MMP3, and TIMP1 in UC patients tissues, enteritis cells and UC mouse model

**Figure 5:**
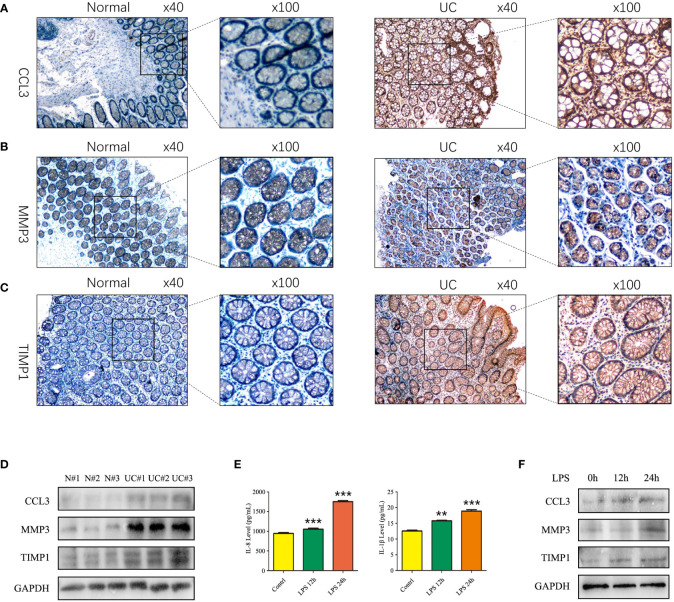
Expression of CCL3, MMP3, and TIMP1 in UC patients and induced enteritis cells. **(A)** The expression of CCL3. **(B)** The expression of MMP3. **(C)** The expression of TIMP1. **(D)** The protein expression levels of CCL3, MMP3 and TIMP1 in UC patients and healthy controls. **(E)** The inflammatory factors IL-8 and IL-1β secretion level after 1 ug LPS stimulated NCM460 cells for 0h, 12h and 24h. **(F)** The protein expression levels of CCL3, MMP3 and TIMP1 in NCM460 cells stimulated with LPS for 0h, 12h and 24h.

**Figure 6:**
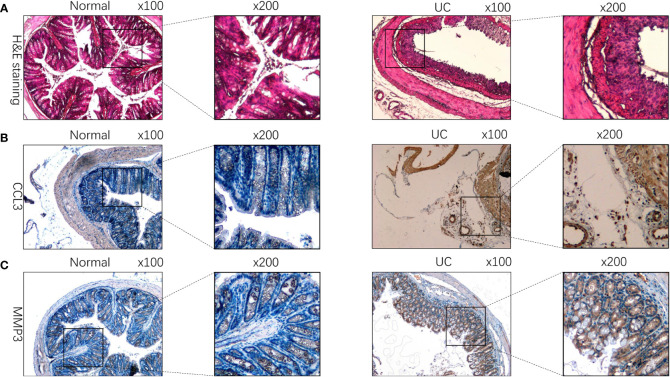
CCL3, MMP3, and TIMP1 expression in DSS-induced colitis mouse model. **(A)** HE staining of tissues of DSS-induced colitis mice and the control group. **(B)** The expression of CCL3 in DSS-induced colitis mouse model. **(C)** The expression of MMP3 in DSS-induced colitis mouse model.

We used IHC to study CCL3, MMP3, and the TIMP1 expression in tissues of healthy controls and patients with UC for verifying the results obtained in patients from the GEO datasets. The results revealed a significant increase in CCL3, MMP3, and TIMP1 expression in tissues of patients with UC patients compared to healthy controls (**Figures 5A–C**). Furthermore, we used western blotting to further verify the expression of CCL3, MMP3, and TIMP1 in the tissues of patients with UC and healthy controls. The results revealed a significant increase in CCL3, MMP3, and TIMP1 expression (**Figure 5D**). We also induced normal colon mucosa NCM460 cells to transform into enteritis cells through LPS, and found that after 24 hours of induction, enteritis cells could secreted higher inflammatory factor IL-1β and IL-8 (**Figure 5E**), and the expression levels of CCL3, MMP3 and TIMP1 in enteritis cells were significantly up-regulated, which were consistent with the expression in the above tissues (**Figure 5F**). Additionally, we determined CCL3 and MMP3 expression in the UC mouse model. **Figures 6A–C** show a significant increase in CCL3 and MMP3 expression levels in tissues of the UC mice compared to the control group.

### 3.5 CCL3, MMP3, and TIMP1 can reflect the therapeutic effect of golimumab (GLM) in UC patients

**Figure 7:**
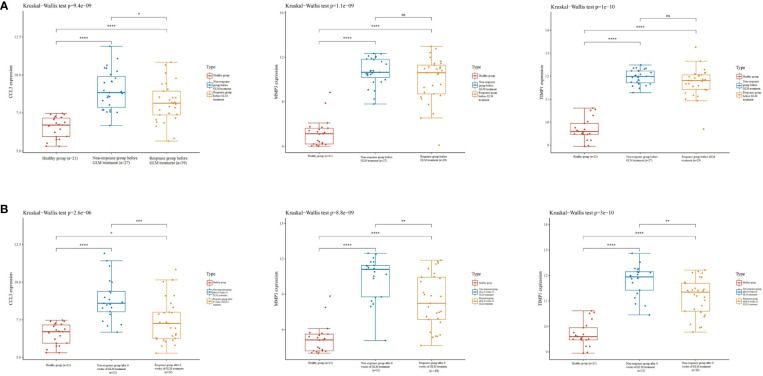
Expression of CCL3, MMP3, and TIMP1 on GLM in patients with UC. **(A)** Expression of CCL3, MMP3, and TIMP1 in the intestinal mucosa of patients with active UC in the non-response and response groups before GLM treatment. **(B)** Expression of CCL3, MMP3, and TIMP1 in the intestinal mucosa of patients with active UC after 6 weeks of GLM treatment in the response and non-response groups.

GLM is a monoclonal antibody that is widely used for treating patients with UC in clinical settings and has been successful in achieving mucosal healing, clinical remission, and response in patients (22). In the GSE92415 (22) dataset, high CCL3, MMP3, and TIMP1 expression levels were observed in the intestinal mucosa of patients with active UC in the GLM response and non-response groups compared to the healthy controls before GLM treatment (**Figure 7A**). In patients treated with GLM for 6 weeks, CCL3, MMP3, and TIMP1 expression levels were increased in the intestinal mucosa of patients with active UC in the GLM response and non-response groups compared to the healthy controls. Furthermore, a significant decrease in CCL3, MMP3, and TIMP1 expression levels was observed in patients in the GLM response group compared to the non-response group (**Figure 7B**). These results suggest that CCL3, MMP3, and TIMP1 expression could be used as indicators to determine the effect of GLM on patients with active UC.

### 3.6 Correlation between CCL3, MMP3, and TIMP1 expression and infiltration of immune cells in colon cancer

**Figure 8:**
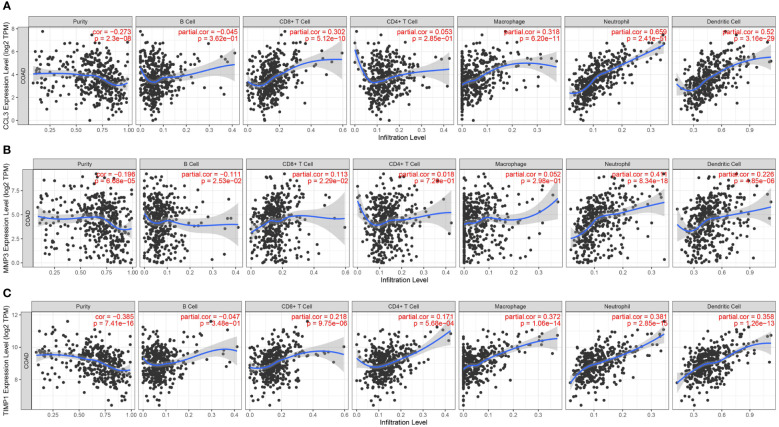
Relationship between CCL3, MMP3, and TIMP1 expression and level of immune cell infiltration in colon cancer. **(A)** CCL3. **(B)** MMP3. **(C)** TIMP1.

Studies have demonstrated that chronic intestinal inflammation increases the risk of developing colitis-associated cancer in patients with UC (23). Hence, we used the TIMER database to analyze the correlation between CCL3, MMP3, and TIMP1 expression and immune cell infiltration status in patients with colon cancer. A significant positive correlation was observed between CCL3 expression and immune cells like CD8+ T cells, macrophages neutrophils, and dendritic cells (DC) (*P* = 5.12e^-10^, cor = 0.302; *P* = 6.20e^-11^, cor = 0.318; *P* = 2.41e^-51^, cor = 0.659; *P* = 3.16e^-29^, cor = 0.52, respectively; **Figure 8A**). MMP3 expression was significantly correlated with immune cells like CD8^+^ T cells, neutrophils, and DCs (*P* = 2.29e^-2^, cor = 0.113; *P* = 8.34e^-18^, cor = 0.411; *P* = 4.85e^-6^, cor =0.226, respectively; **Figure 8B**). Similarly, we observed a positive correlation between TIMP1 expression and the levels of infiltrating CD8^+^ T cells, macrophages, neutrophils, and DCs (*P* = 9.75e^-6^, cor = 0.218; *P* = 1.06e^-14^, cor = 0.372; *P* = 2.85e^-15^, cor = 0.381; *P* = 1.26e^-13^, cor = 0.358, respectively; **Figure 8C**). In addition, CCL3, MMP3, and TIMP1 expression were negatively correlated with tumor purity in colon cancer (P = 2.30e^-8^, cor = -0.273; P = 6.68e^-5^, cor = -0.196 and P = 7.41e^-16^, cor = -0.385, respectively). These results indicate the significance of CCL3, MMP3, and TIMP1 expression in the infiltration of immune cells in colon cancer.

## 4 Discussion

UC is an inflammatory disease of the intestine caused by various factors and is becoming increasingly common. It is characterized by a long clinical course and frequent relapses. Due to the complex nature of UC pathogenesis, there is still a lack of accurate molecular diagnostic biomarkers for patients with UC. Previous studies have shown alterations in the adaptive immune responses as a potential cause of UC. Recent studies have focused on the alterations in innate immune responses in the mucosa of the intestine as the cause of UC pathogenesis (24, 25). However, the underlying mechanisms of UC pathogenesis remain unclear.

In this study, we have analyzed GSE87466, GSE107499, GSE24287, and GSE102133 datasets downloaded from GEO to identify DEGs in patients with UC. GO and KEGG pathway enrichment analyses were performed to identify processes and pathways related to DEGs. CIBERSORT analysis revealed a significant increase in the levels of memory-activated CD4+ T cells, T follicular helper cells, M1 macrophages, and neutrophils in the colon tissues of patients with UC. Finally, we performed comprehensive bioinformatics analysis using Cytoscape to identify CCL3, MMP3, and TIMP1 as hub genes and validated their expression in the UC mouse model.

CCL3, a chemokine, belongs to the CC chemokine family and is involved in immune surveillance and tolerance. It is an effective chemokine for immune cells like lymphocytes and monocytes. Further, CCL3 binds to the receptors like CCR4, CCR1, and CCR5 on immune cells like T cells, DC, B cells, and eosinophils. Various studies have used *in vitro* and *in vivo* models and have shown that CCL3 exerts chemotactic effects on neutrophils and monocytes (26, 27). During monocyte-endothelial cell interactions, monocyte binds to intercellular adhesion molecule-1 and elevates CCL3 levels in these cells. Thus, the levels of CCL3 increase in response to endothelial cell-leukocyte interactions, which is an important mechanism for recruiting cells during inflammatory immune responses (28–30). Banks et al. revealed a correlation between CCL3 expression and severity of inflammation, and an increase in CCL3 expression level was correlated with increased disease activity (31), consistent with our results.

MMP3 belongs to the Zinc-dependent endopeptidase family, primarily secreted by cancer cells, connective tissues, endothelial cells, immune cells like mononuclear macrophages, and neutrophils. Studies have shown the involvement of MMP3 in extracellular matrix degradation (32, 33). MMP3 is a major matrix metalloproteinase detected in the mucosa of patients with IBD (34). Anna et al. showed a correlation between the levels of MMP3 in the serum of children with UC and disease activity. Furthermore, the levels of MMP3 in serum are directly linked to disease activity, wherein an increase in MMP3 levels in serum increases the disease activity. Thus MMP3 could serve as a new marker to determine the activity of UC in children (35). However, TIMP1 can counteract matrix metalloproteinase (MMP) activity. Mounting evidence suggests the involvement of an imbalance in the MMP/TIMP ratio in the pathogenesis of diseases like IBD and cancers (36, 37). Nevertheless, TIMP1 attenuates enhanced MMP activity in IBD (38). Multiple studies have shown an increase in TIMP1 expression in patients with UC. Further, a significant positive correlation was observed between TIMP1 expression and the degree of mucosal injury under an endoscope, C-reactive protein levels, disease, and clinical activity index values (38–40). These results are consistent with our findings.

Our results showed an increase in CCL3, MMP3, and TIMP1 expression levels in the tissues of patients with UC. Further, the CCL3 expression level was higher in patients with active UC compared to patients with inactive UC using GEO datasets. In addition, high CCL3, MMP3, and TIMP1 expression levels were observed in patients with UC and DSS-induced UC mouse models compared to healthy controls or control mice, thereby further validating our results. We performed GSEA, and the results revealed that high expression of CCL3, MMP3, and TIMP1 was related to innate and B cell-mediated immune response, positive regulation of the immune system, T cell activation, and migration. This suggests that CCL3, MMP3, and TIMP1 play an immunomodulatory role in UC pathogenesis. GLM is a monoclonal antibody against TNFα and is involved in regulating the autophagy of cells. It has been widely used for treating adults with UC for several years (41). Our results showed that after 6 weeks of GLM treatment, a significant decrease in CCL3, MMP3, and TIMP1 expression levels was observed in patients with UC in the GLM response group compared to patients with UC in the non-response group. This indicates the involvement of GLM in activating autophagy in UC (42). Recently, studies have focused on the progression of UC to colon cancers. Dysregulation of the immune system and alteration in immune homeostasis in the intestine are important features of the malignant transformation of UCs (43). Therefore, we next analyzed if there was a correlation between the expression of hub genes such as CCL3, MMP3, and TIMP1 and infiltrating immune cells in colon cancer. The results revealed that CCL3, MMP3, and TIMP1 expression was positively correlated with the infiltration status of immune cells like DCs, CD8^+^ T cells, and neutrophils in colon cancer. Together, these results demonstrate the significance of CCL3, MMP3, and TIMP1 in recruiting and regulating infiltrating immune cells in colon cancer.

In summary, we used a combination of bioinformatics tools to identify three hub genes in UC, and their expression was validated in patients with UC and DSS-induced UC mouse models. These results may aid in enhancing our understanding and exploring the mechanisms underlying the pathogenesis of UC. This may also help explore potential biomarkers for diagnosing and treating patients with UC.

## Data availability statement

The datasets presented in this study can be found in online repositories. The names of the repository/repositories and accession number(s) can be found below: http://www.ncbi.nih.gov/geo; https://metascape.org/gp/index.html#/main/step1; http://sangerbox.com/home.html; http://sangerbox.com/home.html; http://string-db.org.

## Ethics statement

The studies involving human participants were reviewed and approved by the Institutional Ethics Committee of Xuzhou central hospital. The patients/participants provided their written informed consent to participate in this study. The animal study was reviewed and approved by the Institutional Ethics Committee of Xuzhou central hospital. Written informed consent was obtained from the owners for the participation of their animals in this study.

## Author contributions

ZP and JF: Conceptualization, Data curation, Formal analysis, Funding acquisition, Investigation, Methodology, Resources, Supervision, Visualization, Writing-original draft, Project administration, Writing-review and editing. HL: Formal analysis, Writing-review and editing. YF and FZ: Data curation, Investigation, Writing-review and editing. FG, GN, and BG: Data curation, Project administration. All authors contributed to the article and approved the submitted version.
